# Comparative Transcriptome Analysis Reveals the Mechanism Related to Fluazinam Stress of *Panonychus citri* (Acarina: Tetranychidae)

**DOI:** 10.3390/insects11110730

**Published:** 2020-10-26

**Authors:** Yi Shang, Yanbo Wang, Jianyu Deng, Xunyue Liu, Yihao Fang, Qiong Rao, Huiming Wu

**Affiliations:** 1 College of Agriculture and Food Sciences, Zhejiang A&F University, Hangzhou 311300, China; yishang2020928@163.com (Y.S.); wyb442806566@163.com (Y.W.); jydeng70@aliyun.com (J.D.); 20150008@zafu.edu.cn (X.L.); 2 Kaihua County Agro-Tech Extension and Service Center, Quzhou 324300, China; fyh645945@163.com

**Keywords:** fluazinam, gene expression differences, transcriptome, *Panonychus citri*

## Abstract

**Simple Summary:**

The citrus red mite, *Panonychus citri*, is an important pest that causes serious citrus production losses in China. The insecticide fluazinam has a good control effect on the pest mites; however, its mechanism of action on mites remains unclear. In this study, we analyzed the transcriptomic sequencing and differential expression genes in *P. citri* treated with fluazinam, and identified some of the genes potential involved in detoxification metabolism related with the fluazinam exposure. Evaluating the efficacy of fluazinam, and analyzing the transcriptome data of *P. citri* under fluazinam stress, potentially provide a new agent for prevention and control of *P. citri*, and also preliminary research results for exploring the mechanism of action of fluazinam on *P. citri*. Given the up-regulated expression levels of genes for Mn-superoxide dismutase and catalase, we speculate that they play an important role in fluazinam-stress action on *P. citri.*

**Abstract:**

The use of a large number of chemical acaricides to control these pest mites has led to an increasing problem of pesticide resistance, which has always been the difficulty in integrated pest management (IPM). Fluazinam has a good control effect on *Panonychus citri*, the serious pest on citrus; however, we only know the mechanism of action of fluazinam as a fungicide and its mechanism of action on mites remains unclear. Through analysis using Illumina high-throughput transcriptomic sequencing and differential expression genes in *P. citri* treated with fluazinam, 59 cytochrome P450 genes, 23 glutathione s-transferase genes, five carboxylate esterase genes, 11 superoxide dismutase genes and 15 catalase genes were identified. The Gene Ontology enrichment and the enrichment of KEGG results showed that the treatment were enrichment for redox enzyme pathways. Evaluating the efficacy of fluazinam, and analyzing the transcriptome data of *P. citri* under fluazinam stress, potentially provide a new agent for prevention and control of *P. citri*, and also preliminary research results for exploring the mechanism of action of fluazinam on *P. citri*. Given the up-regulated expression levels of genes for Mn-superoxide dismutase and catalase, we speculate that they play an important role in fluazinam-stress action on *P. citri.*

## 1. Introduction

*Panonychus citri* (Acarina: Tetranychidae), also known as citrus red mite, is an important fruit tree pest in many citrus-producing areas of China, and can harm many kinds of citrus plants. Chemical control is still the main method to control citrus red mite because of convenience, maneuverability and fast-acting property. However, excessive amounts and frequency of use of chemical pesticides have had many serious consequences [1]. They not only kill the citrus red mite, but also damage its natural enemies, which destroy the ecological balance of citrus orchards and greatly reduce the biodiversity [1,2]. Additionally, some chemical pesticides are difficult to degrade or remain on the surface of fruit, causing food safety and environmental safety problems and harming human health. Studies have shown that resistance of citrus red mite has become increasingly serious, and some acaricides such as pyridaben and propargite no longer provide good control in some areas [3]. This mite has developed resistance to dozens of acaricides, including carbamate, heterocyclic compounds, and new tetronic-acid pesticides, and the problem of pesticide resistance has become increasingly serious [4,5,6,7,8,9]. Therefore, it is very necessary to study the resistance mechanism of citrus red mite, which will also provide a scientific basis for its control and explore the mechanism of action of various new chemical agents.

The fungicide fluazinam is a pyridinamine derivative, mainly used to prevent and control potato late blight and a variety of gray molds, that also has an excellent prevention and treatment effect on diseases caused by such organisms as *Alternaria*, *Phytophthora*, *Sclerotinia*, and *Plasmopara* [10,11]. Previous studies have suggested that fluazinam can be used in the control of pest mites [12], and it was also confirmed to effectively control the citrus red mite in our previous research [13]. This has attracted interest among citrus farmers and pesticide manufacturers, and some areas have registered fluazinam for control of citrus red mite.

Research on the mechanism of action of fluazinam has mainly focused on its inhibition of fungal growth [10,11]. However, how fluazinam acts on arthropods has not been reported. Fluazinam can kill fungi by blocking energy (ATP) synthesis, and can also inhibit the development of invading mycelia and effectively prevent the spread of disease spots, and shows high virulence to growth of mycelia, spore germination and sporulation of wheat pathogen *Fusarium graminearum* [14]. Shao et al. (2015) found that the respiratory rate and glycerin, oxalate and ATP contents of a fluazinam-resistant mutant *Botrytis cinerea* were significantly lower than those of the sensitive strains, indicating that the mechanism of action of fluazinam on *B. cinerea* mainly depends on its inhibition of energy synthesis [15].

Fluazinam is a potent uncoupler, which is metabolically transformed at the level of mitochondria [16], and inhibits mitochondrial complex I activity which can trigger apoptosis [17]. This fungicide can inhibit the oxidative phosphorylation process in cells, so that the energy generated in the respiratory chain cannot be used for phosphorylation of ADP, but can only be consumed as heat energy, thus playing the role of uncoupling [18,19].

The mechanism of action of fluazinam on *P. citri* remains unclear. Based on its inhibition of energy synthesis and the characteristics of uncoupling, the inhibition of ATP synthesis in cells might be an important mechanism for mite control. Transcriptomics is the study of gene transcription and its regulation in vivo. It has played an increasingly important role in the discovery and identification of various functional genes, and it is also a general tool to study resistance mechanisms [20,21,22,23,24]. Transcriptome analysis can help us to explore the impact of fluazinam on the gene expression of *P. citri*, and provide a theoretical basis for the study of the physiological effect and mechanism of fluazinam action on *P. citri*.

In this study, transcriptome sequencing was performed on *P. citri* at different time points after treatment with a sub-lethal concentration of fluazinam, using the Illumina sequencing method. The functional annotation of genes and the differential expression analysis of genes related to fluazinam-stress were also carried out, which provided a scientific basis for exploring the mechanism of fluazinam action on *P. citri*.

## 2. Materials and Methods

### 2.1. P. citri Samples

The *P. citri* were collected from a citrus orchard in Zhejiang Province, and raised on citrus seedlings in a greenhouse in Zhejiang A&F University (25 ± 1 °C, 80 ± 5% RH, 14 h:10 h light:dark). Fluazinam TC was dissolved in acetone solution, and then distilled water was used to prepare the solution of sub-lethal concentration. Samples of active *P. citri* were collected at 6, 24, and 48 h after treatments, with about 300 individuals in each sample. The RNA extraction was then performed for subsequent transcriptome sequencing [25]. Acetone treatment at the same concentration was used as the control. All treatments were replicated three times. All RNA samples were immediately stored in a refrigerator at −80 °C.

### 2.2. RNA Extraction and Library Construction

The total RNA extraction kit of Trizol (UNlQ-10 Column Trizol Total RNA Isolation Kit, Sangon Biotech, Shanghai, China) was used. Sequencing libraries were generated using NEBNext^®^ UltraTM RNA Library Prep Kit for Illumina^®^ (New England BioLabs, Ipswich, MA, USA) according to manufacturer’s protocol. Briefly, magnetic beads with Oligo(dT) were used to concentrate the mRNA. Then fragmentation was carried out using divalent cations under elevated temperature in NEBNext First Strand Synthesis Reaction Buffer. First strand of cDNA was synthesized with random hexamers and M-MuLV Reverse Transcriptase. Both DNA polymerase I and RNase H were used to synthesize the double-stranded cDNA, and then the double-stranded cDNA was purified by AMPure XP beads. The end of the obtained double-stranded cDNA was repaired, a tail was added and the sequencing joint was connected. Then AMPure XP beads were used to screen the size of the segments (preferentially 250–300 bp). Finally, PCR amplification and purification were performed to obtain the final library. Qubit^®^ 2.0 was used for initial quantification and dilution of the library, followed by determination of the insert size of the library. Illumina HiSeq sequencing was performed after the library inspection and 150 bp paired-end reads were generated by Novogene (Beijing, China). The transcriptome raw data has been released already with ID: PRJNA669340.

### 2.3. Assembly Sequencing and Functional Annotation

Full-length transcriptome assembly from RNA-Seq data without a reference genome. Transcriptome *de novo* assembly was carried out using Trinity [26] with min_kmer_cov set to 2 by default and all other parameters set default. The longest cluster sequence was obtained by clustering with Corset hierarchy clustering (https://code.google.com/p/corset-project/) as Unigene for subsequent analysis. The Unigenes were annotated into Nr, Nt, Pfam, KOG/COG, Swiss-Prot, KEGG and GO databases [23,27]. The annotation software and parameters were as follows: Nr: diamond v0.8.22, E-value = 1 × 10^−5^. NT: NCBI blast 2.2.28+, E-value = 1 × 10^−5^. Pfam: HMMER 3.0 package, hmmscan, E-value = 0.01. KOG/COG/Swiss-Prot: diamond v0.8.22, E-value = 1 × 10^−5^. KEGG: KAAS, KEGG Automatic Annotation Server, E-value = 1 × 10^−10^. GO: protein annotation results based on NR and Pfam: software Blast2GO v2.5 and self-written script, E-value = 1 × 10^−6^.

### 2.4. Differential Expression Analysis

The input data of differential gene expression is the read-count data obtained in analysis of gene expression level. We used DESeq R package (1.10.1) for analysis [28], which provides statistical routines for determining differential expression in digital gene expression data using a model based on the negative binomial distribution, and the screening threshold was Padj < 0.05 and |log_2_FoldChange| > 1. In the process of software differences analysis, we corrected the *p*-value using Benjamini and Hochberg’s approach obtained from the original hypothesis test.

### 2.5. Enrichment and KEGG Enrichment Analysis

The GO enrichment analysis method was using GOseq [29] R package, which is based on Wallenius non-central hypergeometric distribution, and can adjust for gene length bias in DEGs. The KEGG database is a resource for understanding high-level functions and utilities of biological systems from molecular-level information [30] (http://www.genome.jp/kegg/). Significant pathway enrichment analysis was conducted with KEGG pathways as units, and hypergeometric tests were used to identify pathways with significant enrichment of differentially expressed genes relative to all annotated genes by using KOBAS 2.0 software [31].

## 3. Results

### 3.1. Splicing Transcript Length Distribution

Transcriptional sequences obtained from Trinity were used as reference sequences for subsequent analysis. The longest cluster sequence was obtained by Corset hierarchical clustering for subsequent analysis. The lengths of transcripts and clustering sequences were statistically analyzed (Table 1).

After splicing, the total number of genes was 28,831, of which 10,492 were 200–500 bp in length, accounting for 36.39% of the total number of genes. There were 8395 genes with a length of 500–1000 bp, accounting for 29.12% of the total number; 4151 genes of length 1000–2000 bp accounted for 14.39%, and 5793 genes of length exceeding 2000 bp accounted for 20.10%.

### 3.2. Gene Annotation Success Rate Statistics

In order to provide comprehensive gene function information, the obtained unigenes were annotated with gene function in seven databases, and the success rate of annotation in the seven databases was calculated (Table 2). In total, 18,771 unigenes (65.1%) were annotated in at least one database.

### 3.3. Unigene Homology Analysis

Homology analysis of unigenes successfully annotated in NR showed that homologs of these genes included multiple species (Figure S1). Of unigenes in the citrus red mite, 48.7% had the highest homology with genes related to the two-spotted spider mite (*Tetranychus urticae* Koch), followed by the brown rice planthopper (*Nilaparvata lugens* (Stal)) with 3.3%. In addition, *Hydra* (Anthoathecatae, Hydridae) and western predatory mite (*Galendromus occidentalis*) had homology with about 2.0%, and 1.6% showed high homology with an amoeba, *Capsaspora owczarzaki*.

### 3.4. GO Function Analysis

After GO annotation of genes, the genes that were annotated successfully were classified according to the next level of the three major categories of GO (Figure S2), Among them, unigenes were divided into cell components, followed by biological processes and finally molecular functions. The three functional groups with the most unigene annotations were, in turn, 8591 unigenes in the cellular processes of the cellular components. There were 8061 unigenes in metabolic process and 7276 unigenes in the bindings in the molecular function. Note that the three functional groups with the least unigenes follow: One unigene in nucleoid in biological process, one unigene in cell aggregation in cell component, and two unigenes in extracellular matrix component in biological process.

### 3.5. KOG Function Analysis

KOG, euKaryotic Ortholog Groups, is a database of eukaryotic organisms based on lineal homologous relationship of genes. The obtained *P. citri* unigenes were compared to the KOG database. There were 8601 successfully annotated unigenes (Figure S3). These unigenes were assigned to 25 different functional families in the KOG database. The three functional families with the most annotated unigenes were “Translation, ribosomal structure, and biogenesis”, and “General function prediction only” and “Posttranslational modification, protein turnover, chaperones (post-translation modification, protein conversion, chaperones)”. These families contained 1247, 1116, and 1048 unigenes, respectively, accounting for 14.49, 12.98, and 12.18% of successfully annotated genes. Note the functional families with the least unigenes were “Defense mechanisms” with 44, “Nuclear structure” with 26 and “Cell motility” with eight, accounting for 0.51, 0.3, and 0.09% of successfully annotated genes, respectively (Figure S3).

### 3.6. Differential Gene Analysis

**Figure 1:**
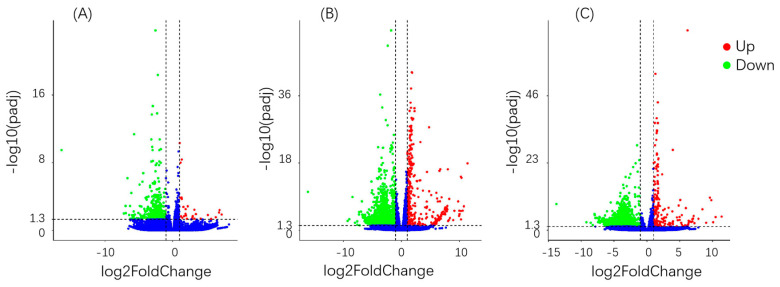
Differential gene volcanoes at different time points. (**A**) at 6 h of fluazinam treatment compare to control, up: 28 genes, down: 487 genes; (**B**) at 24 h of fluazinam treatment compare to control, up: 368 genes, down: 1997 genes; (**C**) at 48 h of fluazinam treatment compare to control up: 191 genes, down: 1381 genes.

Based on the statistics of differentially expressed genes between different treatments and control, a quantitative relationship between the number of differential genes and the total number of genes was obtained. After 6 h of treatment, only 28 differential genes were up-regulated and 487 were down-regulated (Figure 1); after 24 h, 368 were up-regulated and 1997 down-regulated and, after 48 h, 191 were up-regulated and 1381 down-regulated. After 24 and 48 h of treatment, the number of differentially expressed genes was significantly higher than that after 6 h, and the proportion relative to the total number of genes increased significantly. The number of differentially expressed genes was the highest at 24 h after treatment. The number of down-regulated genes in the total gene composition of the three treatment groups, significantly more genes were down-regulated than up-regulated (differential gene screening conditions were |Log2 FoldChange| > 1 and Padj < 0.05).

### 3.7. GO Enriched Gene Analysis

The GO gene enrichment analysis is the application of biological information databases and statistical tools to assign enriched target genes to biological pathways or modules of known functions, thereby allowing in-depth study of gene function from a biological perspective. The purpose is to screen out two groups or enriched gene sets with different expression levels between multiple groups. The 10 functional options with the most significant GO enrichment in different treatment groups were selected for statistical comparison and the results are shown in Table 3. At 6 h, GO enrichment contained the largest number of differential genes: 255 of metabolic process (GO: 0008152) followed by 209 of catalytic activity (GO: 0003824). The most enriched function was fungal-type cell wall (GO: 0009277); however, only six of the differential genes were related to this option. In addition, oxidation–reduction process (GO: 0055114) and oxidoreductase activity (GO: 0016491) also showed some degree of enrichment.

Based on the enrichment of GO function at this time point, we speculate that within a short period of application of fluazinam, the stress response of *P. citri* to fluazinam was manifest by significant changes in metabolic activity and catalytic activity in the body. At the same time, the body’s redox process and energy metabolism were also somewhat affected. At 24 h, GO enrichment statistics showed that the functional item with the highest enrichment level was oxidation–reduction process, followed by oxidation activity, which included the functional item (metabolic process) with the largest number of differential genes. A total of 1059 differential genes were related to the function of metabolic process. Based on the 24-h GO enrichment, we speculate that significant changes in redox process and energy metabolism were closely related to the mechanism of action of fluazinam on *P. citri*. In addition, the abnormal metabolic function may suggest the subsequent response of *P. citri* to fluazinam. The functional item with the highest enrichment level at 48 h was cofactor binding (GO: 0048037), followed by coenzyme binding (GO: 0005319) and oxidation activity (GO: 0016491). Among the 48-h statistical GO enrichment functions, redox was still an important item of enrichment, and cofactor coenzyme binding also showed a high degree of enrichment.

### 3.8. KEGG Enrichment Pathway Analysis

**Figure 2:**
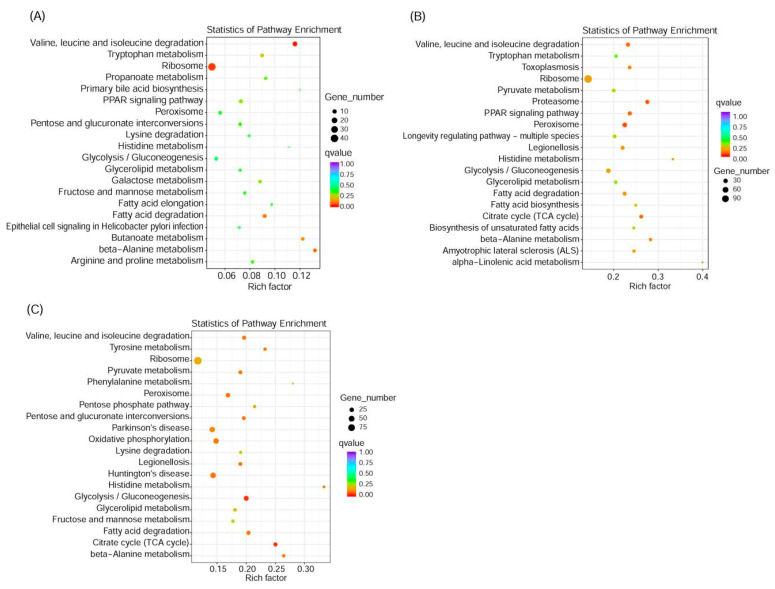
Enrichment scatter diagram of KEGG pathways at different time points. (**A**) at 6 h of fluazinam treatment compare to control, up: 28 genes, down: 487 genes; (**B**) at 24 h of fluazinam treatment compare to control, up: 368 genes, down: 1997 genes; (**C**) at 48 h of fluazinam treatment compare to control, up: 191 genes, down: 1381 genes.

The results of the KEGG enrichment pathway analysis are shown in Figure 2. Compared with GO enrichment results, at 6 h, the KEGG enrichment pathway information showed that the significant results were degradation of valine, leucine and isoleucine degradation, as well as on the ribosome. At 24 h, the KEGG enrichment pathways were proteasome and peroxisome, similar to the abnormal results of redox and energy metabolism found in the GO enrichment function at 24 h. At 48 h, the KEGG enrichment result showed that the enrichment pathway was glycolysis/gluconeogenesis and citrate cycle (TCA cycle) still showed a close relationship with energy synthesis and metabolism in the living body. The GO and KEGG enrichment results indicate that the mechanism of action of fluazinam on *P. citri* was important and associated with its energy metabolism.

### 3.9. Genetic Verification

In order to verify the authenticity of the transcriptome data, a total of 10 genes were randomly selected from three different treatments (6, 24 and 48 h) for qPCR experiments. The overall gene expression trend was consistent with the transcriptome data except two genes (Table 4).

## 4. Discussion

Cytochrome P450, glutathione s-transferase and carboxylate esterase are three important supergene families in the metabolic resistance of insects (and pest mites) [32,33,34]. Ding et al. indicated that P450 gene expression was over-transcribed in acaricide exposures of *P. citri*. The gene *CYP4CF1* is induced by pyridaben and *CYP4CL2* expression is induced by abamectin, azocyclotin, and pyridaben [35]. Transcriptome and differential expression analysis of *Tetranychus cinnabarinus* following treatment with a sub-lethal concentration of β-sitosterol revealed that the detoxification genes of *Tetranychus cinnabarinus* were up-regulated, including 28 carboxyl/cholinesterases and ABC transporter C-class. Some defense-related proteins were also activated, such as toll-like receptors, legumain and serine proteases [36]. In this study, a total of 59 P450-related genes were correlated with fluazinam, among which the CYP2, CYP3, and CYP4 clans were the most widely distributed. These genes are involved in the metabolic pathways of *P. citri* to exogenous substances (ko01100). In addition, 23 glutathione-S-transferase genes and five carboxylesterase genes were identified among the differentially expressed genes. Pathway annotations suggested that these genes might be involved in the metabolic process of *P. citri* in response to exogenous chemicals (ko00982 and ko00983). Therefore, we hypothesize that these genes are closely related to fluazinam stress of *P. citri*.

Many researchers have carried out “omics” studies on mites, including reproduction and sexual differentiation, as well as the detoxification and resistance mechanisms related to acaricides [37,38]. Previous authors considered that the analysis of pest mites at the molecular level based on high-throughput sequencing was helpful to identify the genes related to xenobiotic metabolism [36] and insecticide resistance [39]. Through transcriptome data analysis of *Tetranychus cinnabarinus*, 10 gene categories related to pesticide resistance were identified: P450, GST, CarE, AChE, GluCl, nAChR, GABA receptor, sodium channel, ATPase, and Cyt b genes [39]. Further GO enrichment analysis using data on differentially expressed genes of fenpropathrin-susceptible and -resistant strains of *Tetranychus cinnabarinus* showed that the differences in catalytic activity and binding between the two strains were the most significant, suggesting that these two biological functions might affect the sensitivity of *Tetranychus cinnabarinus* to fenpropathrin [40]. Comparative transcriptome study of resistant and susceptible strains of *P. citri* resulted in identification of 211 metabolism genes and target genes related to insecticide resistance [22]. These conclusions provided a basis and reference for our research. In our study, catalytic activity was significantly enriched and higher than for other GO functional items at 6 h after fluazinam treatment. Thus, it is likely that catalytic activity of mites in vivo changed rapidly after being stimulated by exogenous chemical fluazinam. The genes enriched in this functional term may also be involved in fluazinam-stress in *P. citri*.

In addition, GO and KEGG enrichment analyses showed that the influence of fluazinam on *P. citri* was not only in catalytic activity and metabolic pathway, but also in energy synthesis and redox process. The mechanism of action of fluazinam in other organisms is uncoupling and blocking energy synthesis [41,42]. In this study, a total of 11 superoxide dismutase (SOD) and 15 catalase genes were identified. Our results revealed SOD and catalase genes involved in the mechanism of action of *P. citri* on exogenous fluazinam. The SOD is an active enzyme existing widely in animals, plants and microorganisms, which can remove the harmful substances produced in biological metabolism in the innate immune system [43,44]. The SOD can catalyze the disproportionation of superoxide free-radicals in organisms and eliminate these free radicals. Moreover, SOD can block the damage to cells caused by oxygen free-radicals and repair the damaged cells in time [45]. The SODs can be divided into three types: Cu-ZnSOD, MnSOD, and FeSOD [46]. The Cu-ZnSODs mainly exist in the cytoplasm of eukaryotic cells and are one of the most widely distributed SODs in nature; MnSODs are mainly concentrated in the mitochondria and prokaryotic cells of eukaryotic cells, and FeSODs mainly exist in prokaryotic cells [44]. Much recent research has shown that SOD plays a major role in protecting insects against various environmental stresses [47], including insecticides. Previous studies showed that SOD plays a major role in the process of insect (and mite) response to various adverse environments, including pesticide [48] and temperature stresses [49,50].

The gene encoding a MnSOD (*PcSOD3*) plays a role in the anti-oxidative damage to *P. citri*. Gene cloning and functional exploration of the SOD of *P. citri* showed that MnSOD (*PcSOD3*) in *P. citri* has more strong antioxidant ability than Cu-ZnSOD (*PcSOD1* and *PcSOD2*) [51]. In addition, they also found that after *P. citri* was subjected to external environmental stresses such as acaricides, extreme temperature and ultraviolet radiation, three different types of SOD genes in the body showed up-regulation to varying degrees, with the gene for MnSOD (*PcSOD3*) greatly up-regulated, suggesting that it plays an important role in *P. citri* response to adverse environmental stress [51,52]. Treatment of zebrafish with a half lethal concentration of fluazinam resulted in no significant change in expression levels of genes for Cu-ZnSOD and catalase (CAT), while that for MnSOD significantly increased [53]. In this study, genes for MnSOD and CAT in *P. citri* were significantly up-regulated by fluazinam treatment. The enzyme MnSOD mainly exists in mitochondria and plays an antioxidant role, whereas Cu-ZnSOD exists outside mitochondria. Based on the experimental data, we speculate that fluazinam may promote the generation of a large number of oxidative free-radicals in the mitochondria of *P. citri*, leading to abnormal mitochondrial function and thus playing a role in killing the mite. Oxidative stress is a key factor in the mode of action of pesticide imidacloprid at low doses to *Drosophila*. This pesticide induced increase levels of reactive oxygen species (ROS) to affect mitochondrial function, energy levels, the lipid environment, and transcriptomic profiles [54].

## 5. Conclusions

In this study, 59 cytochrome P450 genes, 23 glutathione s-transferase genes, five carboxylate esterase genes, 11 superoxide dismutase genes and 15 catalase genes related with the fluazinam exposure were identified by transcriptomic analysis and differential expression genes in *P. citri* treated with fluazinam. Given the up-regulated expression levels of genes for Mn-superoxide dismutase and catalase, we speculate that they play an important role in the mechanism of fluazinam action on *P. citri*. Based on the experimental data, we speculate that fluazinam may promote the generation of a large number of oxidative free-radicals in the mitochondria of *P. citri*, leading to abnormal mitochondrial function and thus playing a role in killing the mite, that they play an important role in the mechanism of fluazinam action on *P. citri*. The transcriptome data of *P. citri* are important in revealing the mechanism of acaricidal action of fluazinam, and provide theoretical basis for the control of resistance of mite in IPM.

## Figures and Tables

**Table 1:** List of distribution of stitching length and frequency.

Transcript Length Interval	200–500 bp	0.5–1 kbp	1–2 kbp	>2 kbp	Total
Number of transcripts	18,885	15,594	13,651	29,443	77,573
Number of genes	10,492	8395	4151	5793	28,831

**Table 2:** Success rates for gene annotation.

Databases with Annotation	Number of Genes	Percentage
NR	14,734	51.1
NT	9499	32.94
KO	8018	27.81
Swiss-Prot	12,746	44.2
GO	14,277	49.51
KOG	8601	29.83
All databases	4386	15.21
At least one database	18,771	65.1
Total unigenes	28,831	100

NR: non-redundant protein sequences; NT: nucleotide sequences; KO: KEGG orthology; GO: gene ontology; KOG: euKaryotic ortholog groups.

**Table 3:** GO enrichment analysis for different times.

Time	GO Accession	Description	Term Type	*p*-Value	DEG Item	DEG List
6 h	GO: 0009277	Fungal-type cell wall	cellular component	8.75 × 10^−8^	6	368
	GO: 0003824	Catalytic activity	molecular function	2.69 × 10^−7^	209	368
	GO: 0008152	Metabolic process	biological process	1.36 × 10^−6^	255	368
	GO: 0055114	Oxidation–reduction process	biological process	6.04 × 10^−6^	73	368
	GO: 0016491	Oxidoreductase activity	molecular function	1.08 × 10^−5^	71	368
	GO: 0048037	Cofactor binding	molecular function	1.33 × 10^−5^	34	368
	GO: 0008610	Lipid biosynthetic process	biological process	1.39 × 10^−5^	22	368
	GO: 0003857	3-Hydroxyacyl-CoA dehydrogenase activity	molecular function	1.52 × 10^−5^	9	368
	GO: 0042430	Indole-containing compound metabolic process	biological process	2.48 × 10^−5^	15	368
	GO: 0006550	Isoleucine catabolic process?	biological process	2.60 × 10^−5^	9	368
24 h	GO: 0055114	Oxidation–reduction process	biological process	4.81 × 10^−9^	263	1627
	GO: 0016491	Oxidoreductase activity	molecular function	3.97 × 10^−8^	253	1627
	GO: 0006081	Cellular aldehyde metabolic process	biological process	9.11 × 10^−6^	35	1627
	GO: 0008152	Metabolic process	biological process	1.38 × 10^−5^	1059	1627
	GO: 0009277	Fungal-type cell wall	cellular component	2.01 × 10^−5^	6	1627
	GO: 0044283	Small molecule biosynthetic process	biological process	2.72 × 10^−5^	87	1627
	GO: 0006550	Isoleucine catabolic process	biological process	2.77 × 10^−5^	19	1627
	GO: 0006552	Leucine catabolic process	biological process	2.77 × 10^−5^	19	1627
	GO: 0006574	valine catabolic process	biological process	2.77 × 10^−5^	19	1627
	GO: 0009083	Branched-chain amino acid catabolic process	biological process	2.77 × 10^−5^	19	1627
48 h	GO: 0048037	Cofactor binding	molecular function	1.50 × 10^−14^	103	1128
	GO: 0050662	Coenzyme binding	molecular function	1.75 × 10^−13^	88	1128
	GO: 0016491	Oxidoreductase activity	molecular function	5.25 × 10^−13^	209	1128
	GO: 0008152	Metabolic process	biological process	4.18 × 10^−11^	770	1128
	GO: 0055114	Oxidation–reduction process	biological process	6.79 × 10^−11^	204	1128
	GO: 0072330	Monocarboxylic acid biosynthetic process	biological process	2.45 × 10^−9^	34	1128
	GO: 0003824	Catalytic activity	molecular function	2.77 × 10^−9^	617	1128
	GO: 0032787	monocarboxylic acid metabolic process	biological process	5.14 × 10^−9^	67	1128
	GO: 0016616	oxidoreductase activity, acting on the CH-OH group of donors, NAD or NADP as acceptor	molecular function	9.08 × 10^−9^	43	1128
	GO: 0006550	isoleucine catabolic process	biological process	1.44 × 10^−8^	20	1128

**Table 4:** Transcriptome data validation.

Gene	Annotation	Time	Fold ^a^	Fold ^b^
CYP392A22	Cytochrome P450	6 h	−2.08	−2.43
CYP385C8	Cytochrome P450	6 h	−2.22	−1.56
*Panonychus citri* vitellogenin	Vitellogenin	24 h	6.6	1.19
Catalase	Catalase-like (*Tetranychus urticae*)	24 h	1.24	2.43
Superoxide dismutase 3	Superoxide dismutase 3 (*Panonychus citri*)	24 h	1.02	1.5
Cu.Zn-superoxide dismutase	Cytoplasmic Cu.Zn-superoxide dismutase (*Ditylenchus destructor*)	24 h	1.01	−1.61
*Panonychus citri* heat shock gene	*Panonychus citri* heat shock protein 70-2 mRNA	24 h	1.65	1.5
Superoxide dismutase (*Tieghemostelium lacteum*)	Superoxide dismutase	48 h	−33.3	−20
CYP13A7	Putative cytochrome P450 CYP13A7	48 h	−36.2	−13.74
Carboxylesterase family protein	Carboxylesterase family protein (*Planoprotostelium fungivorum*)	48 h	−8.45	1.06

Note: ^a^: transcriptome sequencing results, ^b^: qPCR detection results.

## Supplementary Materials

The following are available online at https://www.mdpi.com/2075-4450/11/11/730/s1, Figure S1: Unigene homology map (Species classification). Figure S2: GO classification map. Figure S3: KOG classification map.
