# Supplementary material for: Comparative Transcriptome Analysis Reveals the Mechanism Related to Fluazinam Stress of Panonychus citri (Acarina: Tetranychidae)
Source: Insects. 2020 Oct 26;11(11):730. doi: 10.3390/insects11110730 (PMC7692568; doi:10.3390/insects11110730)
Supplement: Supplementary file 1 [file insects-11-00730-s001.pdf]

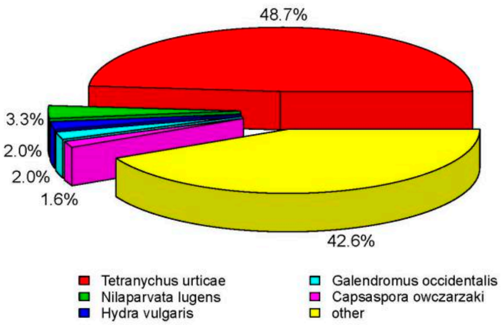

Figure S1. Unigene homology map (Species classification).

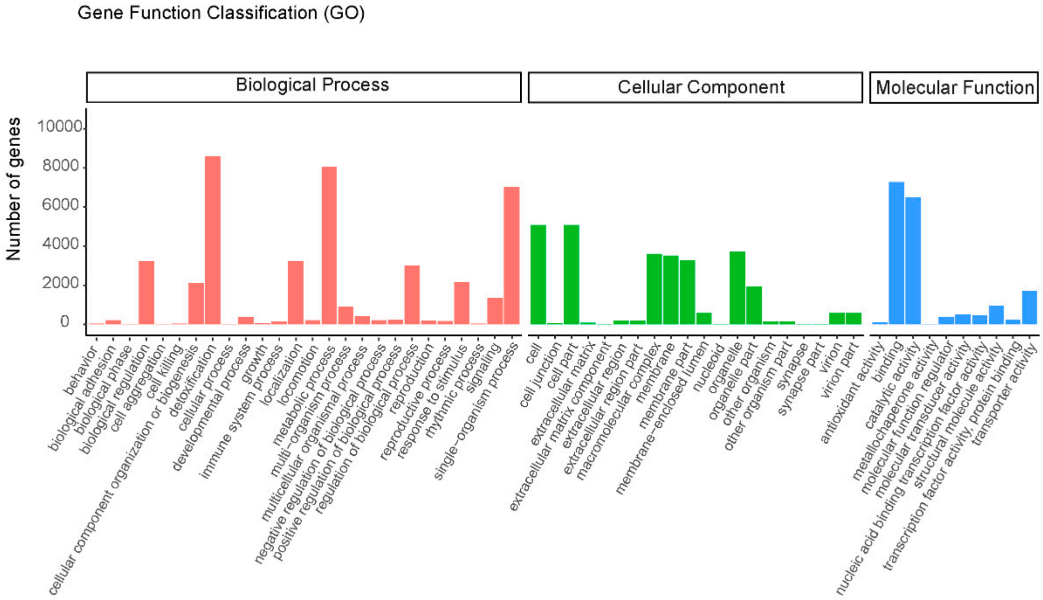

Figure S2. GO classification map

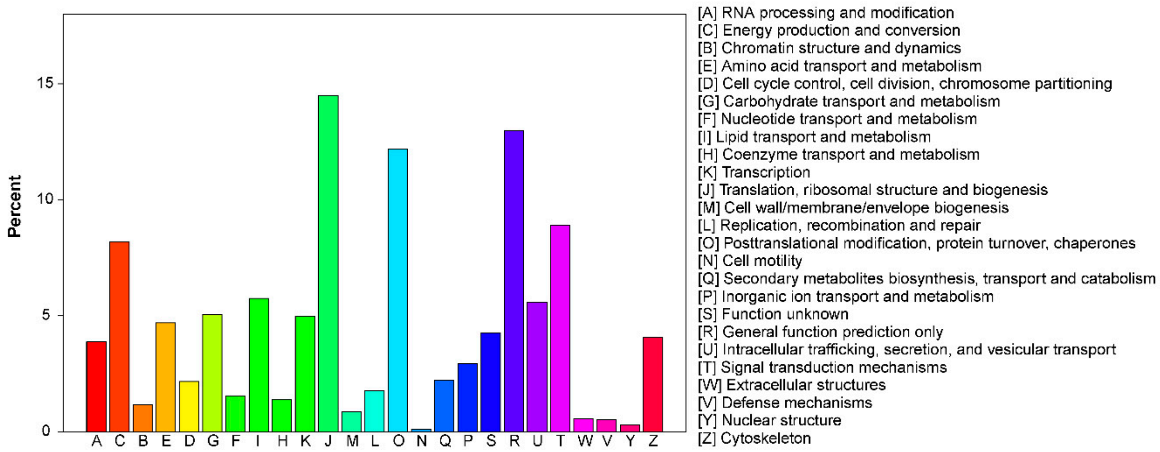

Figure S3. KOG classification map.
